# l‐α‐Lysophosphatidylinositol (LPI) aggravates myocardial ischemia/reperfusion injury via a GPR55/ROCK‐dependent pathway

**DOI:** 10.1002/prp2.487

**Published:** 2019-05-24

**Authors:** Olivia J. Robertson‐Gray, Sarah K. Walsh, Erik Ryberg, Ann‐Cathrine Jönsson‐Rylander, Christopher Lipina, Cherry L. Wainwright

**Affiliations:** ^1^ Cardiometabolic Health Research School of Pharmacy & Life Sciences Robert Gordon University Aberdeen Scotland UK; ^2^ Cardiovascular& Metabolic Disease IMED AstraZeneca R&D Mölndal Sweden; ^3^ Division of Cell Signalling & Immunology School of Life Sciences University of Dundee Dundee Scotland UK; ^4^ Present address: Institute of Cardiovascular & Medical Sciences College of Medical Veterinary and Life Sciences University of Glasgow Glasgow Scotland UK

**Keywords:** cell signaling, GPR55, ischemia/reperfusion injury, lysophosphatidylinositol, rho kinase

## Abstract

The phospholipid l‐α‐lysophosphatidylinositol (LPI), an endogenous ligand for GPR55, is elevated in patients with acute coronary syndrome, and a GPR55 antagonist cannabidiol (CBD) reduces experimental ischemia/reperfusion (I/R) injury. While LPI activates multiple signaling pathways, little is known about which ones are important in cardiomyocytes. In this study we explored whether activation of the Rho kinase/ROCK/p38 MAPK pathway is responsible for LPI‐induced extension of I/R injury. Using a high‐throughput screening method (dynamic mass redistribution; DMR), mouse‐ and human‐induced pluripotent stem cell (iPSC) cardiomyocytes exposed to LPI were shown to exhibit a rapid, sustained, and concentration‐dependent (1 nmol L^−1^‐30 μmol L^−1^) cellular response. Y‐27632 (ROCK inhibitor; 10 & 50 μmol L^−1^) and CBD (1 μmol L^−1^) both abolished the DMR response to LPI (10 μmol L^−1^). In murine iPSC cardiomyocytes, LPI‐induced ROCK and p38 MAPK phosphorylation, both of which were prevented by Y‐27632 and CBD, but did not induce JNK activation or cleavage of caspase‐3. In hearts isolated from wild type (WT) mice subjected to 30 minutes global I/R, LPI (10 μmol L^−1^) administered via the coronary circulation increased infarct size when applied prior to ischemia onset, but not when given at the time of reperfusion. The exacerbation of tissue injury by LPI was not seen in hearts from GPR55^−/−^ mice or in the presence of Y‐27632, confirming that injury is mediated via the GPR55/ROCK/p38 MAPK pathway. These findings suggest that raised levels of LPI in the vicinity of a developing infarct may worsen the outcome of AMI.

**Abbreviations**
CBD: cannabidiol
DMR: dynamic mass redistribution
HEK: human embryonic kidney
iPSC: induced pluripotent stem cell
JNK: c‐Jun N‐terminal kinase
LPI: l‐α‐lysophosphatidylinositol
p38 MAPK: p38 mitogen‐activated protein kinase
PAF: platelet activating factor
RISK: reperfusion injury salvage kinase
ROCK: Rho‐assisted protein kinase
S‐1‐P: sphingosine‐1‐phosphate

## 1 INTRODUCTION

Reperfusion of an occluded artery is vital to the salvage of the myocardium affected by ischemia, however the reintroduction of blood and oxygen paradoxically causes further injury and cardiomyocyte death known as ischemia/reperfusion (I/R) injury.^12^ Immediate “lethal” I/R injury occurs within the first few minutes of reperfusion with the critical early events being an increase in oxidative stress, [Ca^2+^]_i_ overload,^13^ opening of the mitochondrial permeability transition pore (MPTP^15^) and the activation of pro‐apoptotic pathways.^14^, ^26^ It is well established that the release of bioactive lipids from platelets at the site of clot formation during acute myocardial infarction (AMI) contributes to the eventual extent of myocardial I/R injury, with some lipids (eg sphingosine‐1‐phosphate; S‐1‐P) exerting cardioprotective effects (reviewed in 21) and others exacerbating (eg platelet activating factor: 22, 32) I/R injury. Platelets also release the lysophospholipid l‐α‐lysophosphatidylinositol (LPI) and are thought to be the source of the elevated plasma levels of LPI seen in patients undergoing coronary angioplasty for acute coronary syndrome (ACS^27^). LPI acts as an endogenous ligand at the G‐protein coupled receptor GPR55^36^ which is expressed in various rodent^40^ and human^18^ tissues including the cardiovascular system, where it is expressed on vascular smooth muscle and endothelial cells^6^, ^7^ and ventricular cardiomyocytes.^45^, ^52^

Although the functional importance of the GPR55/LPI system in cardiovascular physiology and pathophysiology is poorly understood, we have previously shown that mice lacking GPR55 exhibit moderate systolic dysfunction with age and have a decreased contractile reserve in response to adrenoceptor stimulation,^45^ suggesting a physiological role in maintaining cardiac function. In contrast, observations that GPR55 tissue expression and circulating levels of LPI are raised in conditions where there is heightened cardiovascular risk (such as obesity and metabolic syndrome^33^); that cardiac LPI levels rise during asphyxia‐induced myocardial ischemia,^24^, ^25^ and that the GPR55 antagonist CBD reduces myocardial infarct size in rats in vivo^9^, ^46^ all point to a potential detrimental role for LPI/GPR55 in cardiovascular pathologies such as AMI.

Studies in GPR55‐expressing HEK cells have linked GPR55‐mediated LPI responses to activation of several signaling pathways, including activation of RhoA and ROCK resulting in an increase in intracellular calcium ([Ca^2+^]_i_) and activation of G_α13._ 17 Similarly, LPI induces a rapid and transient increase in [Ca^2+^]_i_ in cardiomyocytes through an action at GPR55 receptors located on both the sarcolemma and the membranes of intracellular organelles,^52^ although any involvement of RhoA/ROCK signaling in the response to LPI in cardiomyocytes remains to be investigated. [Ca^2+^]_i_ overload is a major factor in cardiomyocyte death following myocardial I/R^5^, ^28^ and activation of the RhoA/ROCK pathway plays an important role in the setting of I/R injury as it is activated at the time of reperfusion to suppress the RISK pathway and extend reperfusion injury.^14^ Thus, our underlying hypothesis was that activation of the GPR55/RhoA/ROCK pathway by LPI in the vicinity of the ischemic myocardium, may contribute to exacerbation of I/R injury. To explore this, we initially confirmed in murine and human iPSC cardiomyocytes that activation in response to LPI is mediated by GPR55, utilizing a well‐established label‐free, high‐throughput screening system (Corning^®^ Epic^®^) that measures the dynamic mass redistribution (DMR) of cells and enables real‐time detection of integrated cellular responses in living cells.^41^ This allowed us to make a rapid determination of the optimum concentration and exposure time to LPI for subsequent studies in murine iPSC cardiomyocytes and isolated Langendorff‐perfused hearts, which were undertaken to confirm that stimulation of GPR55 by LPI leads to activation of the RhoA/ROCK/p38 MAPK pathway and to an exacerbation of myocardial injury in cultured cardiomyocytes and isolated hearts, respectively.

## 2 MATERIALS AND METHODS

### 2.1 Dynamic mass redistribution (DMR) in response to LPI

The underlying principle of determining changes in cellular DMR has previously been described in detail.^10^, ^11^, ^41^ To measure DMR, cells are seeded on to a microplate containing a resonant waveguide grating biosensor, which measures changes in the local index of refraction upon mass redistribution within a living cell monolayer grown on the biosensor. When a receptor is stimulated by a ligand, the change in DMR in the cells is manifested as a shift in the wavelength of light that is reflected from the sensor. The magnitude of this wavelength shift is proportional to the total change in the biomass proximal to the sensor surface (amount of DMR) and thus GPCR activation is translated into signaling pathway‐specific optical signatures. This technique has been previously used to demonstrate activation of GPR55 in other cell types,^19^ but not in cardiomyocytes, and has been identified as the preferred assay technique to study GPR55 pharmacology and function since it provides a much more accurate determination of agonist potency. In the present studies, to measure the DMR in response to LPI, murine (Cor.At^®^ CL‐i; source tissue mouse tail tip; miPSC) and human(iCell^®^; 1099441; hiPSC)‐induced pluripotent stem cell (iPSC) cardiomyocytes were seeded (miPSCs—7500 per well; hiPSCs—12,000 per well) in Epic^®^ 384 Well fibronectin coated cell assay microplates using a Multi‐drop Combi cell dispenser (Thermo Electron Corporation, UK) and cultured (37°C in 5% CO_2_) in Cor.At^®^ medium for 7 days (miPSC cardiomyocytes) or 12 hours (hiPSC cardiomyocytes) until 80%‐100% confluent. Post culture, each well was gently washed with buffer (Hank's Balanced Salt Solution; HBSS, 20 mmol L^−1^ 4‐(2‐hydroxyethyl)‐1‐piperazineethanesulfonic acid (HEPES), 0.01% Bovine Serum Albumin (BSA; essentially fatty acid free, ≥96%) and 0.0005% dimethyl sulfoxide (DMSO); pH7.4) using an ELxMicroplate Washer (Bio‐Tek) and the microplates incubated at 26°C for 1 hour. Baseline readings were then taken every minute for a 3‐minute period prior to addition of LPI alone (1 nmol L^−1^‐30 μmol L^−1^), or LPI (10 μmol L^−1^) in the presence of the GPR55 antagonist cannabidiol (CBD; 1 μmol L^−1^), or the ROCK inhibitor Y‐27632 (10 and 50 μmol L^−1^), using a Biomek^®^NM^P^ laboratory automated workstation (Beckman Coulter, Sweden); 100 μmol L^−1^ ATP was used as a positive control. The microplate was then reinserted into the Corning^®^Epic^®^ System and the DMR measurements in each well were recorded at 1‐minute intervals over a 90‐minute time period. To confirm that any effects of Y‐27632 and CBD on LPI responses were not due to a cytotoxic effect, cell viability of miPSC cardiomyocytes exposed to CBD (1 μmol L^−1^) or Y‐27632 (10 and 50 μmol L^−1^) for 90 minutes was determined using an MTT (3‐(4,5‐dimethylthiazol‐2‐yl)‐2,5‐diphenyltetrazolium bromide) assay. Briefly, miPSC cardiomyocytes were seeded at a density of 20,000 cells/well in a 96‐well plate and cultured in Cor.At^®^ media (supplied by the manufacturer) for 7 days at 37°C (in 5% CO_2_). Following treatment with pharmacological agents, media was removed, replaced with MTT (1 mg/mL) and plates incubated for 4 hour at 37°C. The formazan product was then dissolved by adding DMSO to the wells and absorbance measured at 560 nm using a plate reader (Bio‐Tek).

### 2.2 Activation of downstream signaling pathways by LPI in miPSC cardiomyocytes

Activation of ROCK, p38 MAPK, JNK, and caspase‐3 cleavage in response to LPI stimulation was determined in miPSC cardiomyocytes cultured as described in Section 2.1. Experiments were performed with cells grown at a confluency of 70%‐80% and then serum starved overnight (16 hours) prior to experimentation. Following challenge with LPI, with or without the ROCK inhibitor Y‐27632 or the GPR55 antagonist CBD, the cells were lysed and the resulting lysates subjected to SDS‐PAGE and immunoblotting as previously described.^19^ Briefly, following cell treatment, the cells were washed with ice‐cold PBS and then immediately lysed in ice cold lysis buffer (50 mmol L^−1^ Tris/HCl pH 7.4, 0.27 mol L^−1^ sucrose, 1 mmol L^−1^ sodium orthovanadate, 1 mmol L^−1^ EDTA, 1 mmol L^−1^ EGTA, 10 mmol L^−1^ sodium 2‐glycerophosphate, 50 mmol L^−1^ sodium fluoride, 5 mmol L^−1^ sodium pyrophosphate, 1% (v/v) Triton X‐100, 0.1% (v/v) 2‐mercaptoethanol and protease inhibitor (one tablet/50 mL)). Cell debris was removed from crude cell lysates by centrifugation at 3000*g* for 10 minutes at 4°C, and the resulting supernatant used for Western blot analysis. Proteins from cell lysates (30 μg) were fractionated by SDS‐polyacrylamide gel electrophoresis and immunoblotted using anti‐phospho (Ser1366) ROCK2 (Genetex, Irvin, CA, USA), anti‐Actin (MilliporeSigma, St. Louis, MO, USA), anti‐phospho (Thr180/Tyr182) p38 MAPK (Cell Signaling Technology, Danvers, MA, USA), anti‐phospho (Thr183/Tyr185) JNK (Cell Signaling Technology, Danvers, MA, USA), cleaved caspase‐3 (Asp 175) and native ROCK2 (Cell Signaling Technology, Danvers, MA, USA) antibodies. Primary antibody detection was carried out using a horseradish peroxidase‐conjugated anti‐rabbit IgG antibody (New England Biolabs, Hitchin, Herts, UK) and visualized by enhanced chemiluminescence. Resulting band intensities were quantified using ImageJ software (National Institutes of Health, Bethesda, MD).

### 2.3 Isolated heart studies

All studies were performed under a Project License authorized under the UK Animals (Scientific Procedures) Act 1986, conform to the guidelines from Directive 2010/63/EU of the European Parliament on the protection of animals used for scientific purposes and are reported in line with the ARRIVE guidelines.^23^ Male and female wild type (WT) mice (C57BL/6J; JAX background) were purchased from Charles River Laboratories International Inc. (Margate, UK), while homozygous GPR55 knockout (GPR55^−/−^; JAX background) mice were bred in‐house and were routinely genotyped as previously described.^49^ All animals were housed in the University of Aberdeen Medical Research Facility until experimentation at Robert Gordon University. All mice were grouped according to genotype, gender and age and housed in temperature (21 ± 2°C) and humidity (55 ± 10%) controlled rooms with a 12‐hour light/dark cycle (7 am/7 pm). Additionally, mice were housed (according to husbandry guidelines set by the UK Home Office) in groups not exceeding eight, with ad libitum access to water and food pellets and environmental enrichment. All animals (males and females) were aged between 9‐12 weeks (body weights 18‐32 g) at the time of use and were randomly allocated to experimental groups using random number generator software (Stat Trek, UK).

Mice were anesthetized with ketamine (120 mg/kg) and xylazine (16 mg/kg) via intraperitoneal (ip) injection and the heart rapidly excised, the aorta cannulated and the heart mounted onto a Langendorff retrograde perfusion apparatus (AD Instruments Ltd, UK) and perfused with Kreb's Henseleit buffer (119 mmol L^−1^ NaCl, 4.7 mmol L^−1^ KCl, 1.18 mmol L^−1^ KH_2_PO_4_, 2.41 mmol L^−1^ MgSO_4_, 25 mmol L^−1^ NaHCO_3_, 2.52 mmol L^−1^ CaCl_2_ and 10.88 mmol L^−1^ C_6_H_12_O_6_; pH 7.4; 37°C; 2‐2.5 mL/min). Following a 15‐minute stabilization period, since flow to the heart was to be stopped during global ischemia, a slow bolus injection of LPI (500 μL of a 10 μmol L^−1^ solution over a 30 second period) or vehicle (500 μL of 0.1% DMSO) was administered via a side‐port of the aortic cannula 10 minutes prior to 30 minutes of no‐flow global ischemia followed by 30 minutes reperfusion. This concentration of LPI was used to reflect the LPI levels present in the coronary circulation seen in clinical cases of acute coronary syndrome (1‐12 μmol L^−1^), and the DMR and ROCK phosphorylation studies had confirmed that the peak response to LPI develops within 10 minutes and is sustained for >40 minutes. At the end of each protocol, the hearts were frozen (−20°C for 24 hours), sliced into four sections (2‐3 mm thickness) and the third section from the apex stained with 2,3,5‐Triphenyl‐tetrazolium chloride (TTC; 1%) for 30 minutes at 37°C to distinguish between viable and necrotic tissue, fixed in 10% neutral buffered formalin (Formal Fixx^™^) for 2 hours and then photographed with an EOS 1100D camera (Canon, UK) attached to a Leica S4E microscope (Leica Microsystems Ltd, UK). Images were subsequently coded for blinded analysis and infarct size determined via computerized planimetry (ImageJ software) where red and white staining was indicative of healthy and infarcted tissue, respectively.

### 2.4 Statistical analysis

For all experiments, data are expressed as mean ± SEM of n observations. For the ROCK2 phosphorylation assay, n refers to the number of experiments performed using separate cell preparations and data were expressed as a ratio of phospho‐ROCK2 to ROCK2. For the Epic studies, the time courses of the responses were obtained by plotting the DMR activity (arbitrary units) measured at 1‐minute intervals and peak DMR activity was determined from the time course plots and measured at the point where the response reached a maximum before either reaching a plateau or declining; n refers to the number of plates tested for each intervention. For isolated heart studies, n refers to the number of hearts or tissue samples used for the study. Power calculations performed on data from previous studies indicated that to detect a 30% difference in infarct size with 85% power, the minimum group size is 5. Therefore, all experiments employed hearts from at least five animals for each intervention. However, since the studies were performed over several months, contemporaneous control experiments in wild type mice were included throughout to avoid any influence of seasonal variation and therefore the data were pooled, resulting in larger group sizes. Infarct size is expressed as a percentage of total ventricular area of the tissue slice. For both cell studies and isolated heart studies, comparisons were made using either Student's unpaired t‐test (two‐group comparison) or one‐way ANOVA followed by a Bonferroni *post‐hoc* test (multiple comparisons). All statistical analyses were carried out using GraphPad Prism^®^4 software (GraphPad Software, Inc. USA). In all cases a value of *P* < 0.05 was taken to indicate statistical significance.

### 2.5 Drugs and solutions

LPI sodium salt from *Glycine max* (soybean), CID‐16020046, TTC, PBS, BSA, Cor.At^®^ CL‐i cardiomyocytes (miPSC cardiomyocytes) 1M kit with puromycin and Cor.At^®^ medium, HBSS and all the components for Kreb's Henseleit solution (with the exception of NaCl and NaHCO_3_) were all purchased from Sigma Aldrich^®^, UK. Ketamine (Vetalar^™^) and Xylazine (Rompun^®^) were obtained from Pfizer and Bayer Healthcare (both Dublin, Ireland) respectively. Y‐27632 dihydrochloride was purchased from Tocris Bioscience (Abingdon, UK). NaCl, NaHCO_3_, and DMSO were bought from Fisher Scientific, UK. Formal Fixx^™^ was supplied by Thermo Fisher Scientific, UK. HEPES was bought from Invitrogen, UK. Corning^®^ Epic^®^ 384 Well Cell‐Based Assay Microplates were acquired from Corning Life Sciences, UK. Human iPSC Cardiomyocytes (hiPSC cardiomyocytes; iCell^®^ Cardiomyocytes) were acquired from Cellular Dynamics^®^, US.

## 3 RESULTS

### 3.1 Effect of LPI on cultured cardiomyocytes—identification of ROCK as a GPR55 signaling pathway

**Figure 1:**
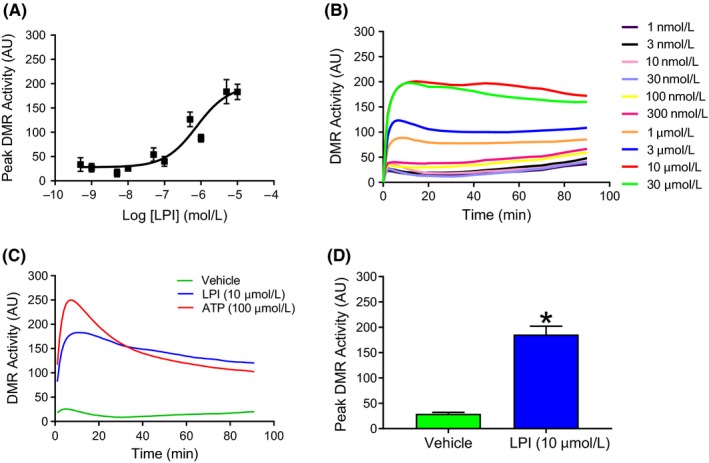
LPI induces a rapid, concentration‐dependent increase in dynamic redistribution (DMR) in murine iPSC cardiomyocytes. LPI (1 nmol L^−1^‐30 μmol L^−1^) induced a concentration‐dependent increase in peak DMR activity (Panel A) that reached a peak within 10 minutes and was sustained for the 90‐minute measurement period (Panel B). ATP (100 μmol L^−1^) was employed as a positive control in all experiments (Panel C). The maximum response seen with 10 μmol L^−1^ LPI was significantly greater than the response to the vehicle (0.1% DMSO; Panel C; *P < 0.05 vs vehicle). Values are shown as mean ± SEM (Panels A & D) or as averaged values (Panels B & C); *n* = 3 experiments performed using different cell preparations. AU = Arbitrary Units

In order to explore whether LPI, acting through GPR55 and ROCK, could have an adverse effect on the outcome of AMI, it was important to initially characterize the responses to LPI in cardiomyocytes. Concentration‐response studies in murine iPSC cardiomyocytes demonstrated that LPI‐induced DMR activity in a concentration‐dependent manner (1 nmol L^−1^‐30 μmol L^−1^; EC_50_ 778 nmol L^−1^; Figure 1A) and that the time course of the DMR response was rapid in onset, reached peak activity at 10 minutes post application and persisted for the duration of the period of measurement (90 minutes; Figure 1B). Since data from clinical cases of acute coronary syndrome have found plasma LPI levels to be in the region of 1‐12 μmol L^−1^,^27^ we chose to use 10 μmol L^−1^ LPI for subsequent experiments since this is in the upper range of clinical data and produced a response of sufficient magnitude and duration (Figure 1C and D) to be sustained throughout the I/R protocol used in the isolated heart studies (70 minutes in total).

**Figure 2:**
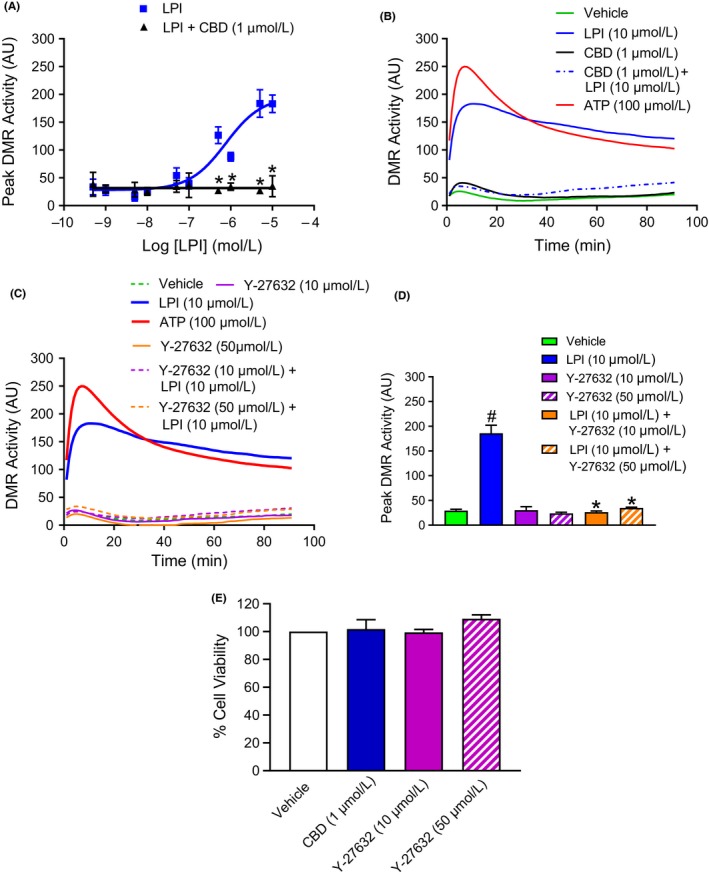
The DMR response in murine iPSC cardiomyocytes is inhibited by the GPR55 antagonist CBD and the ROCK inhibitor Y‐27632. The GPR55 antagonist CBD (1 μmol L^−1^) abrogated the peak DMR response over the full concentration range of LPI (1 nmol L^−1^‐30 μmol L^−1^; Panel A) tested. Both CBD (1 μmol L^−1^; Panel B) and Y‐27632 (10 & 50 μmol L^−1^; Panel C) suppressed all phases of the DMR response to LPI (10 μmol L^−1^) during the 90‐minute measurement period and markedly reduced the peak DMR response to LPI (Panel D). Neither CBD (1 μmol L^−1^) nor Y‐27632 (10 & 50 μmol L^−1^) demonstrated any cytotoxic effects against the cardiomyocytes as determined by MTT assay (Panel E). ATP (100 μmol L^−1^) was employed as a positive control in all experiments measuring DMR. Values are shown as mean ± SEM (Panels A, D, & E) or as averaged values (Panels B & C); n = 3‐4 experiments performed using separate cell preparations for all groups. ^#^ *P *< 0.001 vs vehicle; *P < 0.001 vs LPI alone. AU=Arbitrary Units

**Figure 3:**
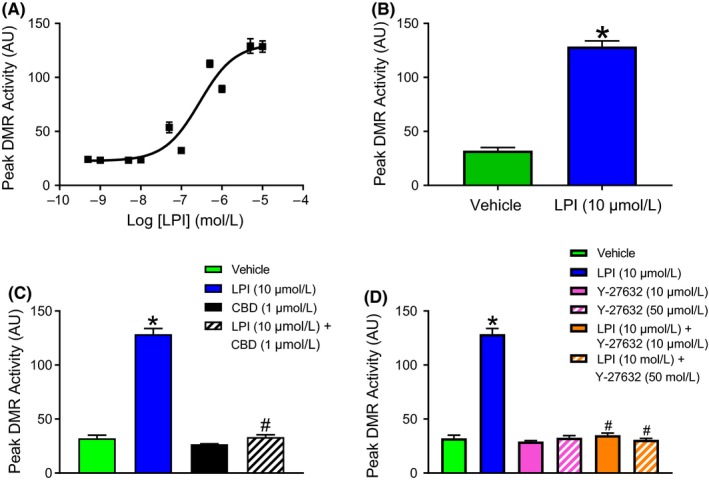
The DMR response to LPI in human iPSC cardiomyocytes is identical to that seen in murine iPSC cardiomyocytes and is similarly inhibited by the GPR55 antagonist CBD and the ROCK inhibitor Y‐27632. LPI (1 nmol L^−1^‐30 μmol L^−1^) produced a concentration‐dependent increase in peak DMR response in human iPSC cardiomyocytes (Panels A & B). Both CBD (1 μmol L^−1^; Panel C) and Y‐27632 (10 & 50 μmol L^−1^; Panel D) markedly reduced the peak DMR response to LPI (10 μmol L^−1^). ATP (100 μmol L^−1^) was employed as a positive control in all experiments. Values are shown as mean ± SEM; n = 4 experiments performed using separate cell preparations for all groups. *P < 0.001 vs vehicle; ^#^ *P *< 0.001 vs LPI alone. AU = Arbitrary Units

Both the peak (Figure 2A) and time‐dependent (Figure 2B) DMR response to LPI in murine iPSC cardiomyocytes were completely prevented by CBD across the full concentration range of LPI, while CBD alone did not induce a cellular response, confirming an action of LPI through activation of GPR55. The ROCK inhibitor Y‐27632 (10 and 50 μmol L^−1^) similarly completely prevented the LPI‐induced increase in DMR activity at all concentrations of LPI (data for 10 μmol L^−1^ shown in (Figure 2C and D), demonstrating that LPI activates ROCK downstream of GPR55 in murine iPSC cardiomyocytes. Neither CBD (10 μmol L^−1^) nor Y‐27632 (10 μmol L^−1^ and 50 μmol L^−1^) had any effect on cell viability (Figure 2E). Finally, to confirm that findings from a murine stem cell‐derived cardiomyocyte line can be translated to humans, the experiments performed in the murine cells were repeated with human iPSC cardiomyocytes and demonstrated that LPI exhibits exactly the same pattern of activity over the same concentration range (EC_50_ 282 nmol L^−1^; Figure 3A and B) and was similarly GPR55 and ROCK‐mediated as shown by inhibition by CBD (Figure 3C) and Y‐27632 (Figure 3D), respectively.

### 3.2 LPI induces ROCK and p38 MAPK phosphorylation in murine iPSC cardiomyocytes

**Figure 4:**
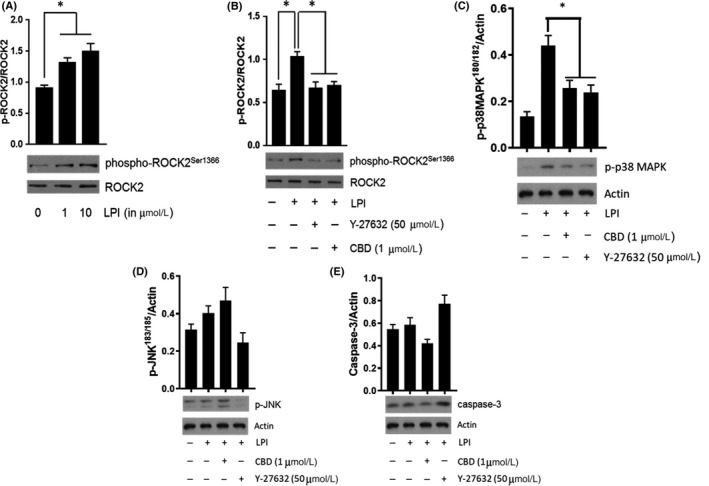
LPI induces ROCK2 and p38 MAPK activation in murine iPSC cardiomyocytes. LPI (1 & 10 μmol L^−1^) produced a concentration‐dependent increase in ROCK2 phosphorylation (Panel A; *P < 0.05 vs control) that was completely inhibited in the presence of both Y‐27632 (50 μmol L^−1^) and CBD (1 μmol L^−1^) (Panel B; *P < 0.05 vs LPI response). LPI also induced p38 MAPK activation that was sensitive to both Y‐27632 and CD (Panel C; *P < 0.05 vs LPI response). In contrast, there was no evidence of significant JNK activation (Panel D) or caspase‐3 cleavage (Panel E) in response to challenge with LPI. Data (mean ± SEM) is expressed as the ratio of phosphorylated to total protein (corrected for protein content); n = 3 experiments performed on separate cell preparations. Western Blots are representative results

To confirm that the changes in DMR in response to LPI were indeed associated with ROCK and p38 MAPK phosphorylation, murine iPSC cardiomyocytes were stimulated with LPI alone (1 & 10 μmol L^−1^ for 10 minutes), or with LPI (10 μmol L^−1^) in the absence or presence of CBD (1 μmol L^−1^) or Y‐27632 (50 μmol L^−1^) prior to Western blotting. As with the DMR studies, LPI‐induced an increase in both ROCK (1 and 10 μmol L^−1^ LPI; Figure 4A) and p38 MAPK (10 μmol L^−1^ LPI; Figure 4C) phosphorylation that was completely abrogated by both CBD (1 μmol L^−1^) and Y‐27632 (50 μmol L^−1^; Figure 4B and C). In contrast, challenging cells with 10 μmol L^−1^ LPI did not result in any significant alterations in either JNK phosphorylation or caspase‐3 activation (Figure 4 D and E).

**Figure 5:**
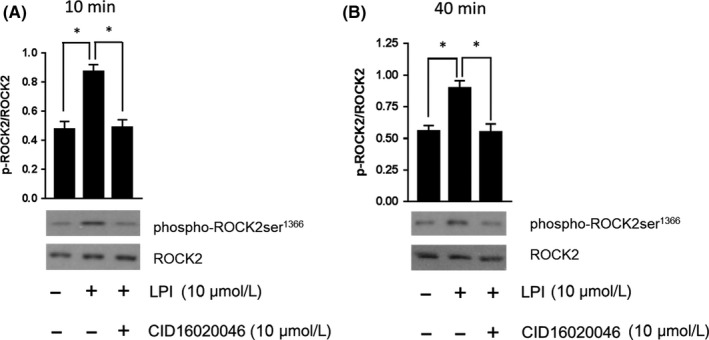
LPI‐induced ROCK2 phosphorylation is a sustained response. Both short (10 minutes; Panel A) and prolonged (40 minutes; Panel B) exposure to LPI demonstrated sustained ROCK2 phosphorylation in murine iPSC cardiomyocytes. A second GPR55 antagonist CID16020046 (10 μmol L^−1^) prevented the response at both timepoints, providing further supporting evidence that the effect of LPI is mediated through GPR55. Data (mean ± SEM) are expressed as the ratio of phosphorylated to total ROCK (corrected for protein content); n = 3 experiments performed on separate cell preparations. Western Blots are representative results. *P < 0.05 vs LPI response

To demonstrate that ROCK activation persists for the duration of global ischemia during the I/R protocol to be used in the subsequent isolated perfused heart experiments, ROCK activation was determined in murine iPSC cardiomyocytes following exposure to LPI (10 μmol L^−1^) for either 10 or 40 minutes. Additionally, since CBD has complex pharmacological activity beyond GPR55 antagonism (eg antagonism at CB_1_ 47; and inverse agonism at CB_2_ receptors,^37^), the role of GPR55 in mediating the responses to LPI was further confirmed in these experiments by using the selective GPR55 antagonist CID16020046 (10 μmol L^−1^). ROCK phosphorylation by LPI was still clearly detectable after 40 minutes (Figure 5A and B) and, as with CBD, CID16020046 completely abrogated the response providing further confirmation that this was mediated through GPR55 activation.

### 3.3 LPI exacerbates I/R injury via activation of GPR55 and ROCK phosphorylation

**Figure 6:**
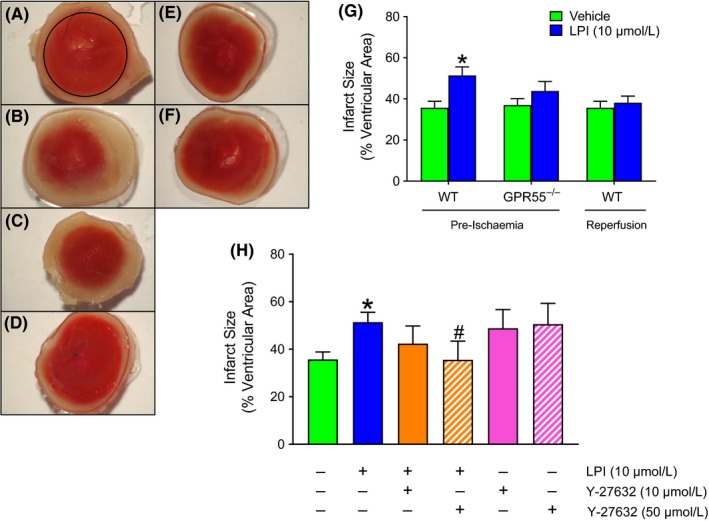
LPI increases myocardial infarct size in hearts from wild type, but not GPR55^−/−^, mouse hearts. Panels A‐F show representative images of TTC (1%) staining of transverse slices of hearts, delineating viable (red) and infarcted (white) tissue, from WT mice exposed to vehicle (A), LPI (10 μmol L^−1^; B) and LPI plus 10 μmol L^−1^ (C) or 50 μmol L^−1^ (D) Y‐27632 prior to 30 minutes global ischemia and 30 minutes reperfusion. Images of hearts from GPR55^−/−^ mice challenged with vehicle (E) or LPI (F) are also shown. Panel G shows that LPI (10 μmol L^−1^) applied 10 minutes before the onset of global ischemia increased infarct size in hearts from WT (n = 14 each for vehicle and LPI), but not GPR55^−/−^ (n = 5 for vehicle and n = 7 for LPI) mice, but not when applied 5 minutes into reperfusion (WT hearts only; n = 9). *P < 0.05 vs vehicle. The higher concentration (50 μmol L^−1^) of the ROCK inhibitor Y‐27632 reversed the effects of LPI‐induced injury in WT hearts (Panel H; n = 5 per group; *P < 0.05 vs vehicle; ^#^ *P *< 0.05 vs LPI alone)

To confirm that LPI was acting through GPR55, hearts from both WT and GPR55^−/−^ mice were used. Challenging WT hearts with LPI prior to the onset of global ischemia significantly increased infarct size compared to vehicle controls (Figure 6A, B and G), whereas this was not seen in seen GPR55^−/−^ hearts, confirming an action of LPI at GPR55 (Figure 6E and F). Interestingly, when LPI was applied to the hearts within the first 1‐2 minutes of reperfusion, there was no increase in infarct size, indicating that the cellular mechanisms that contribute to LPI‐induced injury are activated during ischemia (Figure 6G). When the ROCK inhibitor Y‐27632 (10 and 50 μmol L^−1^; volume delivered 500 μL over 30 second) was applied to WT hearts via the aortic cannula 5 minutes prior to administration of either LPI or vehicle, the LPI‐induced exacerbation of infarct size was abrogated by the higher concentration (Figure 6C, D and H), implicating ROCK in the signaling pathway downstream of GPR55. Interestingly, infarct size did not significantly differ between vehicle‐treated hearts from WT and GPR55^−/−^ mice. This indicates that, at least in isolated hearts, the induction of ischemia does not induce cardiac release of LPI.

## 4 DISCUSSION AND CONCLUSIONS

The data presented from this study has shown for the first time that LPI, a platelet‐derived lysophospholipid that is elevated in acute coronary syndrome, exacerbates the extent of myocardial injury and that, as in other cell types, LPI exerts its cellular responses in cardiomyocytes via activation of the GPR55 receptor and downstream activation of both ROCK and p38 MAPK.

### 4.1 LPI signaling in cardiomyocytes

Despite strong evidence that LPI‐induced RhoA/ROCK signaling is important in other cell types, this pathway has not previously been investigated in cardiomyocytes. Using a high‐throughput (Corning^®^Epic^®^) technique^41^ to measure DMR, which has previously been used to explore cellular responses to GPR55 ligands (including LPI),^19^ we have demonstrated the concentration dependency, time course, and the magnitude of the LPI response in murine and human‐induced pluripotent stem cell‐derived cardiomyocytes. The characteristics of the LPI response in cardiomyocytes are similar to those reported in HEK293 cells, and our demonstration that the LPI response is susceptible to blockade by both CBD and Y‐27632 is consistent with studies in both cancer^3^ and HEK293^17^, ^19^ cells. Crucially, we were able to establish identical responses to LPI, and blockade by CBD and Y‐27632, in both murine and human‐induced pluripotent stem cell cardiomyocytes, establishing that the same signaling pathway is important in both species. In HEK293 cells, GPR55 activation leads to the recruitment of several nuclear transcription factors, all of which activate RhoA leading to phosphorylation of ROCK.^17^, ^19^ In these present studies, we have shown for the first time that exposure of miPSC cardiomyocytes to LPI induces both ROCK and p38 MAPK activation, and that this is dependent upon an action at GPR55.

In cardiomyocytes, one consequence of p38 MAPK activation is the induction of apoptosis,^39^ however we did not see any activation of JNK or caspase‐3 (both markers of apoptosis), suggesting that the increased injury seen in the isolated hearts challenged with LPI prior to I/R was not due to induction of apoptotic cell death but to some other mechanism, such as calcium overload. It has been previously shown that LPI directly increases cardiomyocyte [Ca^2+^]_i_ through activation of both sarcolemmal GPR55 receptors, to stimulate Ca^2+^ entry via L‐type Ca^2+^channels (LTCC) and IP_3_‐mediated Ca^2+^ release from internal stores, and intracellular GPR55 receptors that promote internal Ca^2+^ release via endolysosomal NAADP‐sensitive two‐pore channels.^52^ There is generally scant information on any association between ROCK activation and increases in [Ca^2+^]_i_, and indeed RhoA and Ca^2+^ signaling pathways have been traditionally regarded as being distinct from each other, and so quite how LPI‐induced ROCK activation links to the reported LPI‐induced increases in cardiomyocyte [Ca^2+^]_i_ remains elusive. However, a link between ROCK activation and increases in [Ca^2+^]_i_ has been seen in HEK cells, where a RhoA‐dependent Ca^2+^ signaling pathway mediates LPI‐induced NFAT activation,^17^ suggesting that in some tissues these pathways converge. Indeed, LPI causes a biphasic increase in [Ca^2+^]_i_, in endothelial cells, the first phase of which is PLC‐IP_3_‐dependent and the second phase, ROCK‐dependent.^1^ Therefore, it is feasible that ROCK activation in cardiomyocytes is similarly linked to changes in intracellular calcium.

Although this study has focused on the ROCK/p38 MAPK pathway, LPI has been shown to activate additional signaling pathways on noncardiomyocyte cells, including JNK and AKT/ERK. However, as described above, we did not see any notable JNK activation, and we have found previously in rat neonatal cardiomyocytes that the activation of ERK in response to LPI is very transient (5 minutes) and would therefore be unlikely to account for the sustained response to LPI seen in the DMR studies (Figure S1; unpublished observations).

### 4.2 Mechanisms of LPI‐induced cardiomyocyte injury

Total levels of LPI in the coronary circulation during AMI are significantly elevated (mean 2.95 ± 1.88 μmol L^−1^; range 1‐12 μmol L^−1^), compared to levels seen in either stable angina or no coronary artery disease (mean 1.54 ± 0.99 μmol L^−1^; range 1‐5 μmol L^−1^ 27) and it is believed that the LPI is released from platelets activated at the site of plaque rupture and clot formation, rather than the myocardium *per se*. Platelet‐derived substances released during AMI have ready access (via the coronary circulation) to the cardiac tissue in the vicinity of their release and therefore could influence the injury process within cardiac tissue. By using an isolated perfused heart model, we have demonstrated that application of LPI via the coronary circulation (to mimic exposure to platelet‐derived LPI), has a detrimental influence on the outcome of an ischemia/reperfusion insult, suggesting that patients with elevated coronary LPI levels at the time of an AMI may sustain greater damage to the heart. This action of LPI is most likely to be at the level of the cardiomyocyte, since the use of a global no‐flow ischemia model rules out an adverse (vasoconstrictor) effect of LPI on the coronary circulation during ischemia and, in any case, in *vivo* studies show that LPI causes a depressor rather than a constrictor response.^1^ It is noteworthy that we did not see any difference in the extent of injury between vehicle‐treated WT and GPR55^−/−^ hearts, supporting the notion that there is no endogenous release of LPI from the heart during I/R and that the source of elevated LPI seen during acute coronary events is extra‐cardiac. Indeed, in a separate study (manuscript in preparation), we have found that total LPI levels in WT hearts subjected to I/R are in fact significantly lower than in sham hearts. Our studies also show that administration of LPI at the time of reperfusion does not exacerbate the extent of injury, demonstrating that the LPI‐induced increase in myocardial injury occurs during ischemia.

We have also confirmed that the increase in infarct size following LPI application to isolated hearts is via activation of GPR55 receptors, since infarct size in hearts from GPR55^−/−^ mice was not increased by LPI. Utilizing hearts lacking GPR55, as opposed to the GPR55 antagonist CBD, was a more appropriate way of demonstrating a role for the receptor since, although we know that CBD acts relatively selectively at GPR55 in cell systems, when used in whole tissue or *in vivo* studies it displays a more complex pharmacology.^47^ Moreover, CBD itself reduces infarct size^9^, ^46^ through mechanisms that are not exclusively GPR55‐mediated, and which could have confounded the present studies.

The amelioration of the infarct‐extending effect of LPI in WT hearts by the ROCK inhibitor Y‐27632 supports the notion that cardiomyocyte ROCK activation by LPI is, at least in part, responsible for the exacerbation of I/R injury. ROCK expression is increased in the postinfarct heart^29^ and there is strong evidence that ROCK activation is an important contributor to the expansion of I/R injury, since ROCK inhibition reduces I/R injury through multiple mechanisms including a suppression of endoplasmic reticulum (ER) stress and apoptotic signaling pathways that are activated during reperfusion.^4^, ^29^, ^30^ LPI has been shown (in HEK cells) to induce p38 mitogen‐activated protein kinase (MAPK) downstream of ROCK,^35^ and in the cardiomyocyte activation of MAPK during prolonged ischemia induces a pro‐apoptotic response.^26^ Here we have shown that LPI similarly induces p38 MAPK activation in cultured miPSC cardiomyocytes downstream of ROCK activation, although this is not associated with induction of apoptotic signaling pathways (JNK and caspase‐3). However, p38 MAPK inhibition during I/R has been shown to prevent mitochondrial reactive oxygen species production and Ca^2+^ overload,^42^ pointing to a possible mechanism underlying the LPI‐induced exacerbation of injury.

LPI also promotes Ca^2+^ entry (via LTCCs^52^) into cardiomyocytes, which in the setting of I/R would induce cellular injury through the induction of cardiomyocyte Ca^2+^ overload, activation of mitochondrial K_ATP_ channels and subsequent opening of the mitochondrial permeability transition pore (mPTP^13^). Although an association between LPI‐induced Ca^2+^ entry via LTCC's and activation of ROCK has not been investigated in cardiomyocytes, Ca^2+^ entry via LTCC's has been linked to ROCK activation in vascular smooth muscle cells.^31^ Moreover, in GPR55‐expressing HEK cells LPI activates phospholipase C (PLC) via a ROCK‐dependent pathway,^19^ with the subsequent production of IP_3_ and stimulated Ca^2+^ release from the sarcoplasmic reticulum, which again would cause Ca^2+^ overload. ROCK has also been shown to work in concert with low voltage‐activated T‐type Ca^2+^ channels (Cav3.2 channel), which have also been implicated in Ca^2+^ overload during myocardial ischemia.^34^ Clearly these are potential mechanisms of LPI‐induced cardiomyocyte injury that require further investigation.

Although an investigation of the effect of ROCK inhibition *per se* on infarct size was not a primary aim of the present study, it is interesting that, in contrast to other studies showing an infarct‐sparing effect of ROCK inhibition, we did not see an infarct‐reducing effect of Y‐27632 but rather we observed a slight (not statistically significant) trend toward an increase in infarct size. There are several plausible explanations for this. First, ROCK inhibition has been shown to decrease infarct size and preserve postinfarction cardiac systolic function through various mechanisms including increased collateral blood flow to the myocardium,^51^ improved metabolic status of the ischemic tissue,^16^ reduced myocardial fibrosis,^29^ reduced apoptosis and inflammation^4^ and preservation of the reperfusion injury salvage kinase (RISK) pathway.^30^ Importantly, all of these studies used *in vivo* models and determined end points (inflammation and apoptosis) that occur over extended periods of time (hours to days) after reperfusion or implemented ROCK inhibition at the time of reperfusion.^14^ In contrast, our studies were performed in blood‐free perfused hearts in vitro*,* the hearts were administered Y‐27632 prior to ischemia and relatively short (30 minutes) periods of I/R (when the main mechanisms contributing to cell death are calcium overload and oxidative stress) were used. Consistent with our findings, ROCK activation has been shown to promote cardiomyocyte survival via a PKD pathway^50^ and more recent studies have shown that the two ROCK isoforms (ROCK‐1 and ROCK‐2) play opposing roles in the heart, with ROCK‐1 protecting against cardiac dysfunction and oxidative stress and ROCK‐2 promoting these.^43^ Since Y‐27632 is a nonselective inhibitor that has similar affinities for both ROCK isoforms,^2^ this could explain why in vehicle‐treated hearts, where I/R‐induced ROCK‐1 activation predominates and serves a pro‐survival role, Y‐27632 had a slightly deleterious effect, while in LPI‐stimulated hearts where ROCK‐2 is activated (as shown from our cardiomyocytes studies), it prevented the ROCK‐2 mediated extension of injury.

Off‐target actions of Y‐27632 for ROCK could also potentially account for our findings since, when screened against a panel of kinases it has been shown to inhibit protein kinase‐2 (PRK2^8^; PKC epsilon (PKCε) and protein kinase N1 (PKN).^2^ The expression of PRK2 is very low in the heart,^38^ making it an unlikely target, but PKN is a pro‐survival kinase expressed in response to I/R injury^44^ while PKCε is a known trigger of delayed preconditioning.^20^, ^48^ Since our model did not employ a preconditioning protocol, inhibition of PKN would be the most likely alternative mechanism of the infarct‐extending effect of Y‐27632.

**Figure 7:**
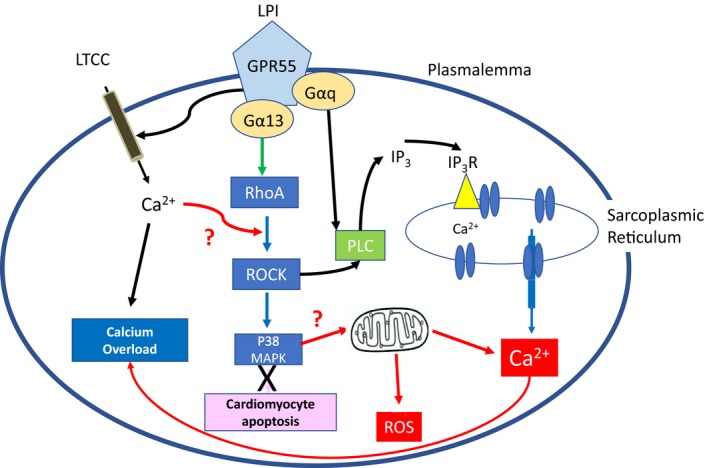
Proposed signaling pathway for LPI in cardiomyocytes that contributes to exacerbation of I/R injury. LPI is known to increase intracellular calcium in cardiomyocytes via direct entry through LTCC's and through PLC/IP_3_‐mediated release from the sarcoplasmic reticulum (black arrows). Here we have shown that LPI results in both ROCK and p38 MAPK activation (blue arrows); while p38 MAPK activation has been linked to a pro‐apoptotic effect during ischemia (pink), lack of evidence of activation of pro‐apoptotic pathways suggests this is not the cause of LPI‐induced cardiomyocyte cell injury. The proposed pathways (red arrows) are p38 MAPK‐induced mitochondrial release of Ca2+ and ROS production

In summary, we present the novel finding that LPI activates murine and human‐induced pluripotent stem cell cardiomyocytes via a GPR55/RhoA/ROCK/p38 MAPK‐dependent pathway, and that this same pathway is responsible for the exacerbation of injury seen in hearts challenged with LPI prior to an ischemia/reperfusion insult. While the precise signaling pathways that ultimately result in a greater injury response following LPI challenge remain to be elucidated, based upon the knowledge of signaling pathways in other cell types we hypothesize (Figure 7) that LPI activates ROCK both directly and indirectly (via Ca^2+^ entry), which (i) further increases [Ca^2+^]_i_ through IP_3_‐mediated release from intracellular stores and (ii) activates p38 MAPK to induce mitochondrial ROS production and Ca^2+^ overload, which together result in enhanced tissue injury.

Of significant clinical application is our demonstration that LPI, at clinically relevant concentrations, induces activation in human‐induced pluripotent stemcell cardiomyocytes through precisely the same mechanism as that seen in murine cells. When taken alongside reports that LPI levels are increased in the coronary circulation in patients with ACS, our data implicate LPI as a contributor to the extent of myocardial injury in the clinical setting.

## AUTHOR CONTRIBUTIONS

OR‐G (PhD student) performed the isolated perfused heart studies, supervized by CW (Principal Supervisor), SW (infarct studies), A‐C J‐R and ER (Epic studies), and undertook all data analysis. CL performed the Western Blotting studies in murine iPSC cardiomyocytes and SW performed the MTT assays. CW obtained the funding from the MRC in collaboration with ACJ‐R, and SW secured the funding from Tenovus Grampian. OR‐G prepared an initial draft of the manuscript, CW produced the final version, and all contributing authors had editorial input to the final version.

## DISCLOSURES

None.
