# Supplementary figures and images for: l‐α‐Lysophosphatidylinositol (LPI) aggravates myocardial ischemia/reperfusion injury via a GPR55/ROCK‐dependent pathway
Source: Pharmacol Res Perspect. 2019 May 24;7(3):e00487. doi: 10.1002/prp2.487 (PMC6533556; doi:10.1002/prp2.487)

Supplementary Figure 1

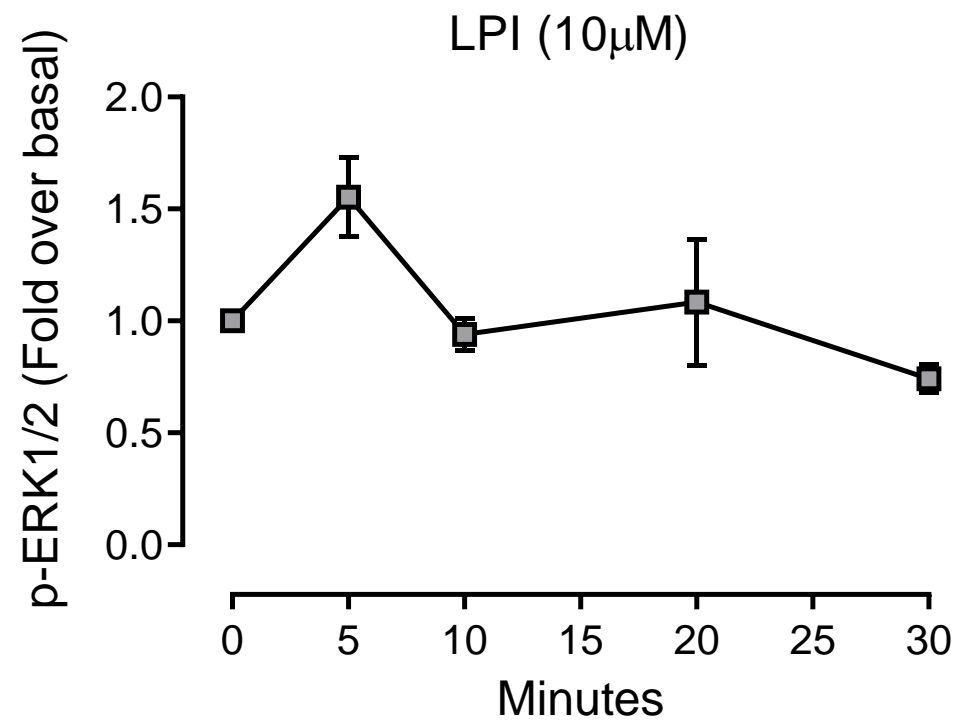

Supplement: Supplementary file 1 [file PRP2-7-e00487-s001.pdf]
